# AKT Regulation of ORAI1-Mediated Calcium Influx in Breast Cancer Cells

**DOI:** 10.3390/cancers14194794

**Published:** 2022-09-30

**Authors:** Alice Hui Li Bong, Trinh Hua, Choon Leng So, Amelia A. Peters, Mélanie Robitaille, Yin Yi Tan, Sarah J. Roberts-Thomson, Gregory R. Monteith

**Affiliations:** 1 School of Pharmacy, The University of Queensland, Brisbane, QLD 4102, Australia; 2 Mater Research, Translational Research Institute, The University of Queensland, Brisbane, QLD 4101, Australia

**Keywords:** breast cancer, AKT regulation, calcium signaling, PTEN, GCaMP6, ORAI1

## Abstract

**Simple Summary:**

A remodeling in calcium homeostasis and the protein kinase AKT signaling pathway often promotes tumorigenic traits in cancer cells. Changes in calcium signaling can be mediated through altered expression or activity of calcium channels and pumps, which constitute a class of targetable therapeutic targets. Currently, the interplay between the two signaling pathways in breast cancer cells is unclear. A better understanding of the association between calcium and AKT signaling, and the molecular players involved may identify novel therapeutic strategies for breast cancers with abnormal AKT signaling. Using fluorescence calcium imaging and gene silencing/knockout techniques, we showed that increased AKT activation results in increased calcium entry, and that this is mediated through ORAI1 calcium channels. Future studies exploring therapeutic strategies to target PTEN-deficient or hyperactivated AKT cancers should consider this novel correlation between AKT activation and ORAI1-mediated calcium influx.

**Abstract:**

Although breast cancer cells often exhibit both abnormal AKT signaling and calcium signaling, the association between these two pathways is unclear. Using a combination of pharmacological tools, siRNA and CRISPR/Cas9 gene silencing techniques, we investigated the association between PTEN, AKT phosphorylation and calcium signaling in a basal breast cancer cell line. We found that siRNA-mediated PTEN silencing promotes AKT phosphorylation and calcium influx in MDA-MB-231 cells. This increase in AKT phosphorylation and calcium influx was phenocopied by the pharmacological AKT activator, SC79. The increased calcium influx associated with SC79 is inhibited by silencing AKT2, but not AKT1. This increase in calcium influx is suppressed when the store-operated calcium channel, ORAI1 is silenced. The results from this study open a novel avenue for therapeutic targeting of cancer cells with increased AKT activation. Given the association between ORAI1 and breast cancer, ORAI1 is a possible therapeutic target in cancers with abnormal AKT signaling.

## 1. Introduction

Breast cancer is the most common cancer in women and second leading cause of cancer-related deaths [1]. Breast cancer cells often possess aberrant cell signaling mechanisms that contribute to their cancerous phenotype. One such pathway commonly dysregulated in breast cancer is the phoshoinositide-3-kinase (PI3K)/AKT pathway [2,3]. Cancer cells with increased activation of the PI3K pathway often have either overexpression/mutation of upstream receptor tyrosine kinases (e.g., human epidermal growth factor receptor), or loss-of-function of the negative PI3K regulator/tumor suppressor, PTEN. This often culminates in increased expression or activation of AKT. AKT is a major regulator of important cellular processes including proliferation, survival and migration. As such, its dysregulation can promote the hallmarks of cancer such as resistance to cell death and uncontrolled proliferation. AKT has three isoforms, AKT1, AKT2 and AKT3, each with a homologous serine/threonine protein kinase site. The specific roles of each isoform in cancer cells have been unclear, although recent studies suggest that these isoforms possess non-redundant functions and specific activation mechanisms (reviewed in [4]). For example, in transgenic mice with activated Akt1 or Akt2 expression within the mammary gland, AKT1 is predominantly associated with tumor initiation, whereas AKT2 is involved in tumor progression and metastasis [5]. AKT inhibitors such as MK2206 are being trialed clinically in breast cancer patients with PTEN loss or mutations; however, benefits appear modest, partially due to dose-limiting toxicities [6]. A comprehensive understanding of the intricacies of the AKT signaling pathway and its interplay with other signaling pathways may unravel more effective therapeutic strategies.

In addition to the PI3K/AKT signaling pathway, calcium ion (Ca^2+^) homeostasis is also often dysregulated in cancer cells. Changes in cytosolic Ca^2+^ control various cellular processes including apoptosis, proliferation, gene transcription and cellular energy production (reviewed in [7]). Ca^2+^ signaling is regulated through the orchestrated actions of specific Ca^2+^ channels, pumps and sensor proteins. As such, a remodeling in expression or activity of these Ca^2+^ regulating proteins can disrupt normal cellular homeostatic processes and result in key tumorigenic traits (reviewed in [7,8]). In non-excitable cells including breast cancer cells, Ca^2+^ influx mainly occurs through store-operated Ca^2+^ entry (SOCE) [9,10,11,12]. During SOCE, release of stored Ca^2+^ in the endoplasmic reticulum results in a conformational change of the sensor protein STIM1. STIM1 then activates ORAI channels on the plasma membrane to mediate Ca^2+^ influx to replenish intracellular Ca^2+^ stores. ORAI1 levels are greater in breast cancer cells of the basal molecular subtype [13,14] and is associated with increased cancer cell invasion and migration [15].

The cellular Ca^2+^ signaling machinery can also be hijacked by oncogenic proteins during tumor transformation and progression. For example, the Bcl-2 family of anti-apoptotic proteins can promote cancer cell resistance to apoptotic stimuli via modulating the activity of inositol 1,4,5-triphosphate (IP_3_) receptors (IP_3_R) (reviewed in [16]). Increased AKT activity confers cancer cell resistance to apoptotic stimuli through IP_3_R phosphorylation, reducing endoplasmic reticulum Ca^2+^ release [17,18]. More recently, Marchi et al. showed that AKT promotes phosphorylation of the regulatory mitochondrial protein MICU1, and this increases basal mitochondrial Ca^2+^ levels leading to tumor progression in various cancer cell lines [19]. There is also some evidence that AKT phosphorylation is dependent on and/or increases Ca^2+^ influx in some cancer cells, such as melanoma, ovarian and prostate cancer cells [20,21,22]. For example, silencing of TRPM4, and in turn reduced Ca^2+^ influx in PC3 and LNCaP prostate cancer cell lines is associated with a decrease in basal AKT1 phosphorylation and reduced proliferation [23]. As such, focussing on Ca^2+^ channels that are associated with AKT activity in specific cancer cell types is a new strategy to target cancer cells with increased constitutive AKT activity.

Currently, there are relatively few studies assessing the link between AKT activity and Ca^2+^ signaling in breast cancer. Previous studies by our group have shown that the PTEN-deficient MDA-MB-468 breast cancer cell line exhibits high levels of basal AKT phosphorylation, and increased basal Ca^2+^ influx compared to the PTEN-functional MDA-MB-231 breast cancer cell line [24,25]. However, it remains unknown whether this increased basal Ca^2+^ influx in breast cancer cells is a direct consequence of high basal AKT activity. In addition, the Ca^2+^ channel mediating possible AKT-sensitive Ca^2+^ influx has not been identified. In this study, we used PTEN-functional MDA-MB-231 breast cancer cells stably expressing the genetically encoded Ca^2+^ indicator GCaMP6m (GCaMP6m-MDA-MB-231) as a model to assess the direct consequence of PTEN loss on Ca^2+^ signaling. We also investigated the link between increased AKT phosphorylation and Ca^2+^ signaling using a novel pharmacological AKT activator SC79. Increased AKT phosphorylation as a result of both PTEN silencing and AKT pharmacological activation resulted in increased Ca^2+^ influx in MDA-MB-231 cells. Furthermore, this increased influx was mediated through the ORAI1 calcium channel. The findings from this study implicate a role for the ORAI1 calcium channel in mediating AKT-regulated Ca^2+^ influx.

## 2. Materials and Methods

### 2.1. Cell Culture

Clonal MDA-MB-231 cells stably expressing the GCaMP6m Ca^2+^ indicator (GCaMP6m-MDA-MB-231) and *ORAI1* knockout GCaMP6m-MDA-MB-231 (*ORAI1*KO) cells were developed as previously described [26,27]. Experiments involving *ORAI1*KO cells also included a non-clonal GCaMP6m-MDA-MB-231 variant as described previously [26]. Parental MDA-MB-231 cells used in our laboratory were purchased from American Type Culture Collection. All cell lines used were authenticated by short tandem repeat profiling at QIMR Berghofer (Brisbane, Australia) using an Agilent Bioanalyzer 2100. Cells were cultured in Dulbecco’s Modified Eagle Medium (DMEM) supplemented with 10% fetal bovine serum and 4 mM L-glutamine in a 37 °C, 5% CO_2_ humidified incubator. Hygromycin (400 µg/mL) was included in the culture media for clonal GCaMP6m-MDA-MB-231 cells. Cells were tested bi-annually for mycoplasma infection using the MycoAlert^TM^ Mycoplasma Detection Kit (Lonza) and cells were used in experiments for no more than six passages.

### 2.2. siRNA Transfection

siRNAs were transfected 24 h after cell seeding at a concentration of 100 nM using DharmaFECT4 (0.1 µL per well; GE Healthcare-Dharmacon). The following SMARTpool ON-TARGETplus siRNAs (Dharmacon) were used: Non-targeting (NT) Control pooled siRNAs (D-001810-10), PTEN siRNA (L-003023-00), ORAI1 siRNA (L-014998-00), AKT1 siRNA (L-003000-00) and AKT2 siRNA (L-003001-00). siRNA knockdown of these targets was confirmed with immunoblotting.

### 2.3. Antibodies and Immunoblotting

Cells were lysed using a tris-based protein lysis buffer containing protease (cOmpleteTM Mini Protease Inhibitor Cocktail) and phosphatase (PhosSTOP^TM^) inhibitors (Roche). A Bradford assay was performed to determine the protein concentrations using the Bio-Rad Protein Assay Dye Reagent (Bio-Rad Laboratories). Gel electrophoresis was performed using a Bio-Rad Mini-PROTEAN^®^ Tetra Cell 4-Gel System, and protein samples were transferred onto a PVDF membrane using the Trans-Blot Turbo Transfer System. Membranes were blocked using 5% skim milk in 0.1% PBST for 1 h and incubated with primary antibodies overnight at 4 °C (1:1000 dilution), except β-actin, which was incubated for 1 h at room temperature (1:10,000 dilution). Goat-anti-rabbit and goat anti-mouse HRP-conjugated secondary antibodies (Bio-Rad Laboratories) were incubated for 1 h at room temperature (1:10,000 dilution). The following primary antibodies were purchased from Cell Signaling Technology: anti-PTEN (CST9559), phospho-AKT (Ser473) (CST4051), total AKT (CST9272), AKT1 (CST2938), AKT2 (CST3063). ORAI1 antibody (4284) was purchased from ProSci Incorporated. β-Actin was purchased from Merck Sigma. Blots were imaged using the Bio-Rad ChemiDoc Touch Imaging System. Protein density was quantified using the Bio-Rad ImageLab software (version 5.2.1) and individual protein bands were normalized to corresponding β-actin band densities.

### 2.4. Fluorescence Imaging of Intracellular Ca^2+^ Signaling

Cytosolic Ca^2+^ changes were imaged using a Fluorescence Imaging Plate Reader (FLIPR^TETRA^, Molecular Devices, San Jose, CA, USA). GCaMP6m-MDA-MB-231 cells were plated in black-walled 96-well microplates at 4000 cells per well and WT and *ORAI1*KO cells were plated at a density of 10,000 cells per well. To assess the effect of siPTEN on cytosolic Ca^2+^ concentration ([Ca^2+^]_CYT_) changes, Ca^2+^ imaging was performed 96 h post-transfection. Briefly, media was removed, and cells were washed twice with physiological salt solution (PSS) containing nominal Ca^2+^ (no added CaCl_2_) before a 15 min incubation with HEPES-buffered PSS nominal at room temperature. Reagents containing BAPTA (100 µM) (Invitrogen) and the Ca^2+^-mobilizing agonists ATP (Merck-Sigma) or trypsin (Merck-Sigma) were then added. For assessment of unstimulated Ca^2+^ influx, 1.8 mM CaCl_2_ was added. To assess the effect of AKT activation on Ca^2+^ influx, cells were pre-incubated with SC79 (Merck-Sigma) for 1 h prior to imaging. To assess SOCE, cells were first pre-incubated in PSS nominal for 15 min, followed by consequent additions of BAPTA (100 µM) and then cyclopiazonic acid (CPA; 10 µM) (Merck-Sigma) to deplete Ca^2+^ stores. DMSO (0.1%) was included as a control for CPA. CaCl_2_ (1.8 mM) was then added to the cells to allow SOCE. Changes in fluorescence (F) were measured at 470–495 nm excitation and 515–575 nm emission wavelengths. Fluorescence normalized to baseline fluorescence (F/F_0_) values were exported from the ScreenWorks (Molecular Devices) software into Microsoft Excel. Relative [Ca^2+^]_CYT_ changes were represented as normalized fluorescence change over time calculated using the formula: F/F_0_–1 (ΔF/F_0_).

### 2.5. Statistical Analysis

Statistical analyses were performed using GraphPad Prism 9.0 (GraphPad Software, Version 9.3.1). Statistical tests used for individual experiments were described in the corresponding figure legends. Unless otherwise specified, all data shown in figures are derived from three biological replicates (n = 3). For all Ca^2+^ imaging experiments, each n value represents the mean of three technical replicates, i.e., three individual wells. *p* values of less than 0.05 were considered statistically significant.

## 3. Results and Discussion

### 3.1. PTEN Silencing Alters Cytosolic Ca^2+^ Signaling in MDA-MB-231 Cells

**Figure 1:**
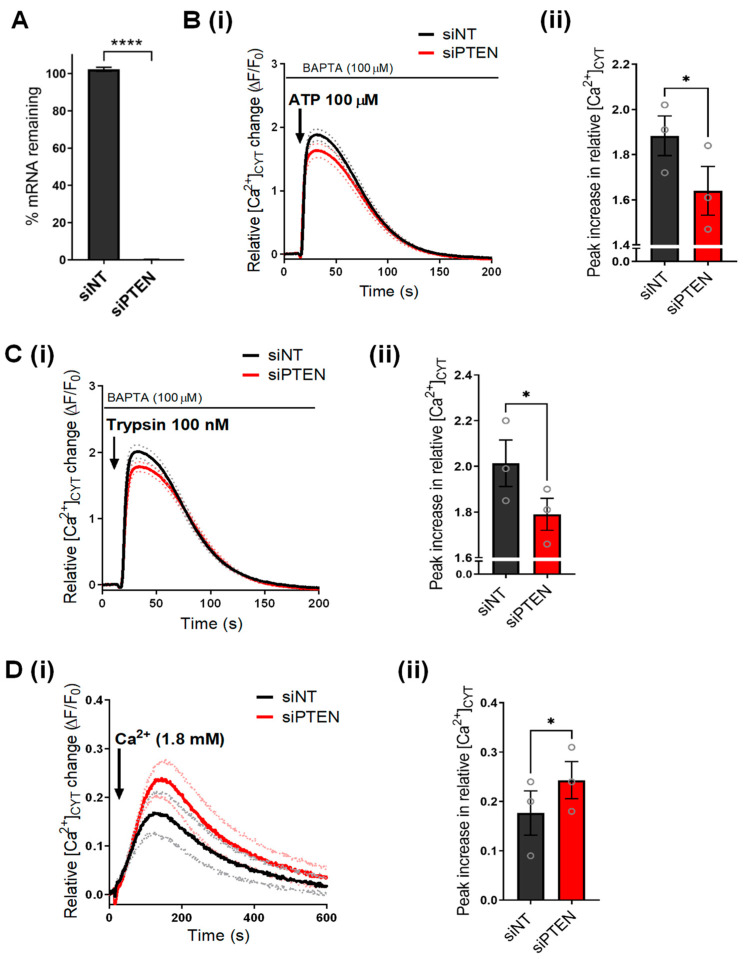
PTEN silencing remodels Ca^2+^ signaling in GCaMP6m-MDA-MB-231 cells. (**A**) Bar graph shows percent mRNA remaining in PTEN silenced (siPTEN) cells compared to NT control (siNT). Traces (i) show the mean relative [Ca^2+^]_CYT_ increases in GCaMP6m-MDA-MB-231 cells as a result of (**B**) ATP 100 µM, (**C**) trypsin 100 nM or (**D**) CaCl_2_ (1.8 mM) addition to siNT or siPTEN-treated GCaMP6m-MDA-MB-231 cells. Dotted lines on the traces represent the S.E.M of the mean F/F_0_ of three biological replicates. Bar graphs compare the (ii) maximal or peak relative increase in [Ca^2+^]_CYT_ between siNT and siPTEN transfected cells. Data represent the mean ± S.E.M. (n = 3) and were analyzed using a paired *t*-test. **** < *p* < 0.0001, * *p* < 0.05.

To investigate the role of AKT on Ca^2+^ signaling in basal breast cancer cells, we first characterized the effect of silencing PTEN, the upstream AKT regulator, on Ca^2+^ signaling in GCaMP6m-MDA-MB-231 cells. We confirmed that PTEN mRNA levels were significantly reduced with PTEN siRNA (siPTEN) (Figure 1A). To assess the effect of PTEN silencing on endoplasmic reticulum Ca^2+^ signaling, ATP, which activates purinergic receptors, and trypsin, an activator of protease-activated receptors, were added to the cells in the absence of extracellular Ca^2+^ [28]. As shown in Figure 1B, PTEN silencing reduced the Ca^2+^ increase induced by ATP (100 µM) addition compared to non-target siRNA control (siNT). PTEN silencing also reduced Ca^2+^ release as a result of trypsin (100 nM) addition (Figure 1C). These results suggest that PTEN silencing either suppresses endoplasmic reticulum Ca^2+^ release or reduces the levels of stored endoplasmic reticulum Ca^2+^. To assess whether siPTEN affects the levels of stored endoplasmic reticulum Ca^2+^, we assessed the Ca^2+^ increase associated with inhibition of endoplasmic reticulum Ca^2+^ reuptake using CPA. We found no significant change in CPA-mediated Ca^2+^ increase as a result of siPTEN (Figure S1). Indeed, studies show that the loss of PTEN expression and/or increased AKT phosphorylation in cancer cells reduces endoplasmic reticulum Ca^2+^ release through the IP3R. This may be mediated via an inhibition of IP3R activity or increased proteasomal degradation of IP3R [18,25,26,29,30]. Next, we assessed the effect of PTEN silencing on unstimulated Ca^2+^ influx in GCaMP6m-MDA-MB-231 cells. As shown in Figure 1D, siPTEN augmented basal Ca^2+^ influx, evident from the significant increase in peak cytosolic free Ca^2+^ ([Ca^2+^]_CYT_) (Figure 1D(ii)) in GCaMP6m-MDA-MB-231 cells with the addition of extracellular Ca^2+^. These results are consistent with an increased basal Ca^2+^ influx previously observed in the PTEN-deficient MDA-MB-468 cells [25].

### 3.2. Increased Ca^2+^ Influx Mediated by PTEN Silencing Is Phenocopied with a Direct AKT Activator

**Figure 2:**
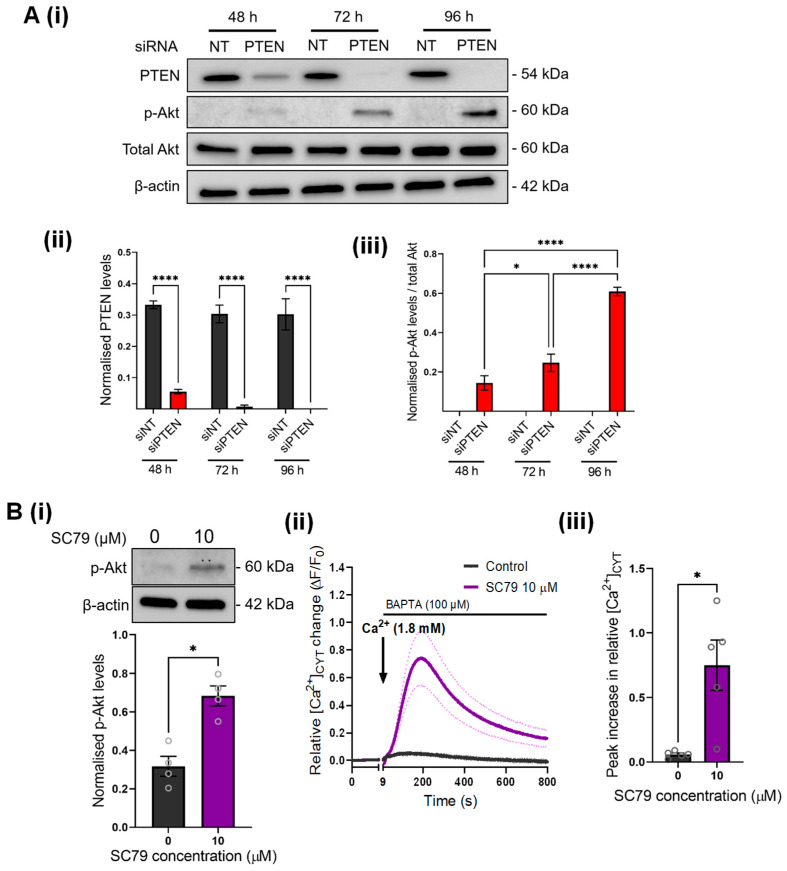
PTEN silencing and SC79 promote AKT phosphorylation in GCaMP6m-MDA-MB-231 cells and increase Ca^2+^ influx. (**A**) (i) Immunoblot shows the effect of siPTEN on protein levels of PTEN and phosphorylated AKT (p-Akt) at 48, 72 and 96 h post-transfection. Bar graphs compare (ii) PTEN and (iii) p-Akt levels in siNT and siPTEN treated cells at 48, 72 and 96 h post-transfection. Statistical analyses were done using a repeated measures one-way ANOVA with Bonferroni’s multiple comparisons test. **** *p* < 0.001; * *p* < 0.05. (**B**) (i) Representative immunoblot and bar graph showing the effect of SC79 (10 µM) 1 h incubation on p-Akt levels. (ii) Trace shows the mean relative [Ca^2+^]_CYT_ increase in GCaMP6m-MDA-MB-231 cells incubated with SC79 (10 µM) for 1 h. (iii) Bar graph compares the mean peak relative increase in [Ca^2+^]_CYT_ between 0 µM and 10 µM SC79 treatments. Statistical analyses for (**B**) were performed using paired t-tests (n = 5). * *p* < 0.05. Uncropped Western blots in Figure S4.

Next, we wanted to confirm that the Ca^2+^ signaling changes observed with PTEN silencing are indeed associated with increased AKT activation. We first confirmed that PTEN silencing promotes AKT activation in GCaMP6m-MDA-MB-231 cells. PTEN silencing resulted in a time-dependent decrease in PTEN protein levels, and this was associated with a significant increase in AKT phosphorylation (Figure 2A). No effect on total AKT protein levels was observed (Figure 2A(ii)). Maximal AKT phosphorylation was observed at 96 h post-PTEN silencing, which corresponded to complete knockdown of PTEN protein levels (Figure 2A(ii)).

AKT is endogenously activated via a PIP3-mediated mechanism involving AKT recruitment to the plasma membrane, where it is phosphorylated by the kinases PDK1 and mTORC2 (reviewed in [31,32]). PTEN counters this pathway via dephosphorylating PIP3 to PIP2. Given that PIP2/PIP3 levels on the plasma membrane can also affect the activity of Ca^2+^ influx channels [33,34], we used SC79, a direct pharmacological activator of AKT, to define the direct link between AKT activity and Ca^2+^ influx [35]. As shown in Figure 2B, SC79 (1 h treatment) significantly increased Akt phosphorylation in MDA-MB-231 cells. To investigate whether the increased Ca^2+^ influx observed in PTEN-silenced cells may be attributed to increased AKT phosphorylation, we assessed Ca^2+^ influx in cells treated with SC79. As shown in Figure 2B(ii), SC79 treatment significantly promoted Ca^2+^ influx. Our results suggest that increased AKT activation increases Ca^2+^ influx in breast cancer cells. This is in contrast to many studies showing that AKT activation is often preceded by increased cytosolic Ca^2+^, such as in ovarian cancer cells and osteoblasts [21,36]. However, a small number of studies using cardiomyocytes have instead shown that AKT activity is associated with increased Ca^2+^ influx, through increased stabilization of L-type Ca^2+^ channels at the plasma membrane [37,38]. Further studies will be required to explore the mechanism by which AKT increases Ca^2+^ influx in breast cancer cells.

### 3.3. SC79-Induced Ca^2+^ Influx Is Inhibited by AKT2 but Not AKT1 Silencing

**Figure 3:**
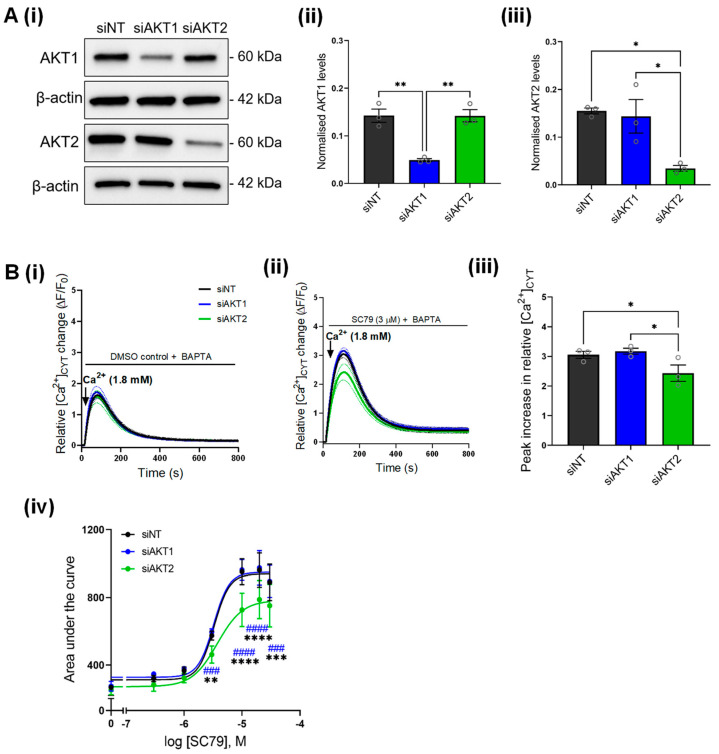
Effect of AKT1 and AKT2 silencing on SC79-mediated Ca^2+^ influx. (**A**) (i) Immunoblot shows the protein levels of AKT1 and AKT2 with siAKT1 and siAKT2 compared to siNT control. Bar graphs show the densitometry analysis of (ii) AKT1 and (iii) AKT2 protein levels as a result of siAKT1 and siAKT2 treatment in GCaMP6m-MDA-MB-231 cells. Statistical analyses were done using a one-way ANOVA with Bonferroni’s test. ** *p* < 0.01, * *p* < 0.05. (**B**) Traces show the effect of AKT1 (siAKT1) and AKT2 (siAKT2) silencing on (i) basal Ca^2+^ influx and (ii) SC79-mediated Ca^2+^ influx (3 µM) in GCaMP6m-MDA-MB-231 cells. (iii) Bar graph show the mean ± S.E.M. of the peak relative Ca^2+^ increase induced by SC79 (3 µM) in cells transfected with siAKT1 and siAKT2 compared to siNT. Statistical analysis was performed using a one-way ANOVA with Bonferroni’s test. * *p* < 0.05. (iv) Concentration-response curve comparing the effect of siAKT1 and siAKT2 on area under curve as a result of SC79-mediated Ca^2+^ influx. Data points on the curves were normalized to the DMSO control for each biological replicate. Statistical analysis was performed using a repeated measures two-way ANOVA with Bonferroni’s test. * denotes statistical difference between siAKT2 and siNT; # denotes statistical difference between siAKT1 and siAKT2. **** *p* < 0.0001; *** *p* < 0.001, ** *p* < 0.01. Uncropped Western blots in Figure S4.

SC79 is a pan-AKT activator, which promotes the phosphorylation of all AKT isoforms [35]. Given that AKT plays isoform-specific roles in cancer cells, and there are currently a lack of studies assessing isoform-specific effects of AKT on Ca^2+^ signaling, we assessed if specifically silencing the AKT1 or AKT2 isoforms had any effect on SC79-mediated Ca^2+^ influx. We first confirmed that AKT1 and AKT2 silencing reduced the protein expression of AKT1 and AKT2, respectively (Figure 3A). AKT1 and AKT2 siRNA did not reduce the low level of basal Ca^2+^ influx in MDA-MB-231 cells in the absence of AKT activation compared to siNT (Figure 3B(i)). Likewise, when cells were treated with SC79, Ca^2+^ influx in cells treated with siAKT1 were not significantly different to cells transfected with siNT (Figure 3B(ii)). However, when AKT2 was silenced Ca^2+^ influx induced by SC79 was suppressed (Figure 3B(iii,iv)), suggesting that AKT2 is the predominant isoform responsible for augmenting Ca^2+^ influx as a result of AKT activation in MDA-MB-231 breast cancer cells.

### 3.4. Increased Ca^2+^ Influx Associated with AKT Activation Is Mediated through ORAI1

**Figure 4:**
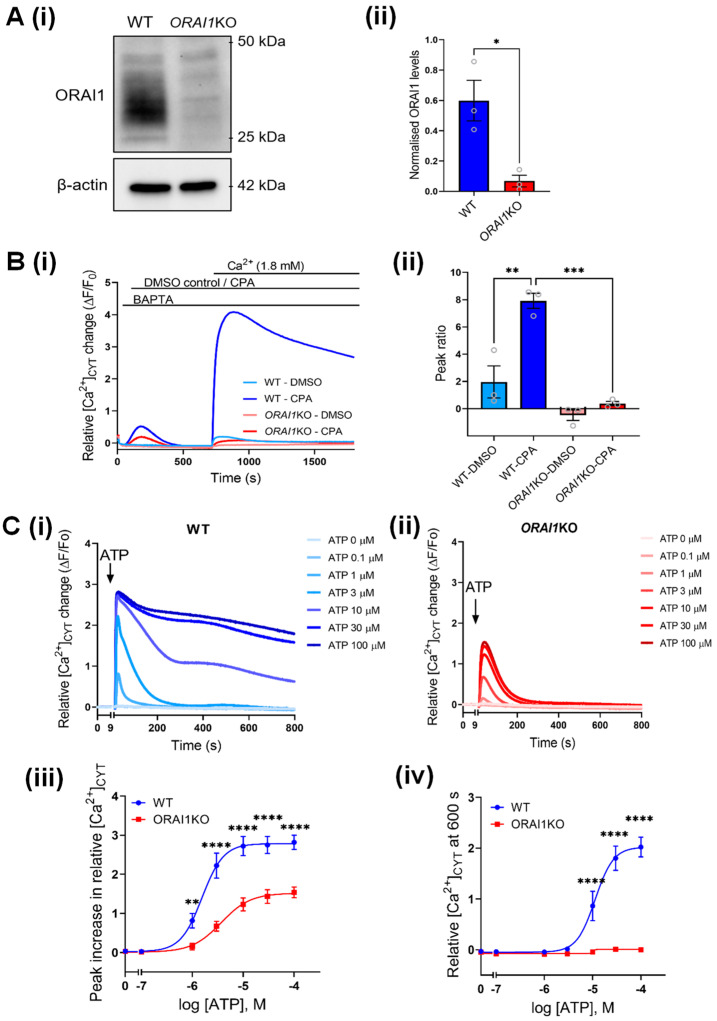
Characterization of ORAI1KO-GCaMP6m-MDA-MB-231 (ORAI1KO) cells. (**A**) (i) Representative immunoblot showing ORAI1 protein levels in GCaMP6m-MDA-MB-231 (WT) and ORAI1KO-GCaMP6m-MDA-MB-231 (ORAI1KO) cells. (ii) Densitometry comparing the levels of ORAI1 protein in WT and ORAI1KO cells. Statistical analysis was performed using a paired t-test. * *p* < 0.05 (**B**) (i) Trace shows Ca^2+^ influx (CaCl_2_ addition) following a 690 s pre-incubation with DMSO (unstimulated Ca^2+^ influx) and CPA (SOCE) in WT and ORAI1KO cells. Bar graphs show the mean ± S.E.M of the ratio of peak 2 (Ca^2+^ re-addition or SOCE) relative to peak 1 (CPA addition). Statistical analysis was performed using a one-way ANOVA with Bonferroni’s test. *** *p* < 0.001; ** *p* < 0.01 (**C**) Traces show mean [Ca^2+^]_CYT_ increase as a result of ATP addition in (i) WT and (ii) ORAI1KO cells. Concentration-response curves compare the (iii) peak relative increase in [Ca^2+^]_CYT_ and (iv) sustained increases in [Ca^2+^]_CYT_ at 600 s between WT and ORAI1KO cells treated with SC79. Statistical analyses were performed using a two-way ANOVA with a Bonferroni’s test. **** *p* < 0.0001, ** *p* < 0.01. Uncropped Western blots in Figure S4.

Higher AKT2 expression and/or activity is associated with the acquisition of metastatic capabilities including tumor cell migration and invasion in breast cancer [5,39,40]. Increased Ca^2+^ influx through ORAI1 is also associated with increased breast cancer cell migration and metastasis in vivo [15]. We thus investigated the possibility that AKT-mediated Ca^2+^ influx in our cells occurs through ORAI1. To do this, we used ORAI1KO-GCaMP6m-MDA-MB-231 (ORAI1KO) cells (Figure 4A) previously generated [26] using CRISPR/Cas9 gene editing. We first confirmed that these ORAI1KO cells were deficient in SOCE stimulated with CPA treatment, compared to parental GCaMP6m-MDA-MB-231 cells (WT) (Figure 4B). As expected, the ORAI1KO cells also exhibited lower Ca^2+^ increases as a result of CPA treatment (Peak 1) compared to WT cells. This reflects lower levels of stored Ca^2+^ in the endoplasmic reticulum, which is expected since the store-refilling mechanism is impaired in these cells [41]. We further assessed whether these ORAI1KO cells could produce physiologically meaningful Ca^2+^ responses by treating these cells with different concentrations of ATP. Both WT (Figure 4C(i)) and ORAI1KO (Figure 4C(ii)) cells exhibited concentration-dependent increases in the [Ca^2+^]_CYT_ peak with ATP addition, with peak [Ca^2+^]_CYT_ increases lower in ORAI1KO. Compared to WT cells, where sustained Ca^2+^ elevations (i.e., SOCE) were observed at 10 µM ATP and above (Figure 4C(i)), ORAI1KO cells had no sustained Ca^2+^ increases at any ATP concentration (Figure 4C(ii)). The initial peak [Ca^2+^]_CYT_ increases were also lower in ORAI1KO cells at 0.1–100 µM ATP concentrations, suggesting that in MDA-MB-231 cells at least, the size of the endoplasmic reticulum Ca^2+^ store is highly dependent on ORAI1-mediated SOCE. This confirms that while these cells are deficient in SOCE, Ca^2+^ signaling is not generally impaired.

**Figure 5:**
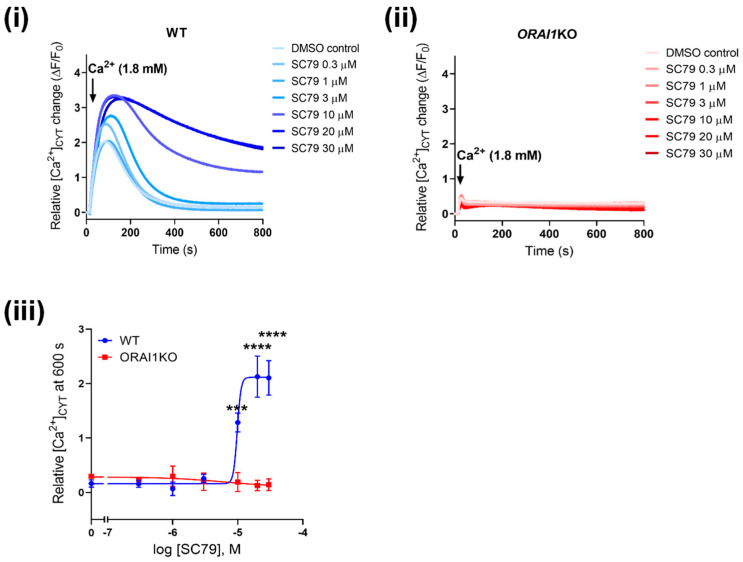
SC79-mediated Ca^2+^ influx is abolished in ORAI1KO-GCaMP6m-MDA-MB-231 cells. Traces show Ca^2+^ influx as a result of SC79 treatment (1 h) in (i) WT and (ii) *ORAI1*KO cells. (iii) Concentration-response curve compares the sustained increases in [Ca^2+^]CYT at 600 s between WT and *ORAI1*KO cells. Statistical analysis was done using a two-way ANOVA with a Bonferroni’s test. **** *p* < 0.0001, *** *p* < 0.001.

Next, we investigated the effect of AKT activation using SC79 on Ca^2+^ influx in ORAI1KO cells. As shown in Figure 5(i), SC79 induced a concentration-dependent increase in Ca^2+^ influx in WT-GCaMP6m-MDA-MB-231 cells. However, in ORAI1KO cells, Ca^2+^ influx induced by SC79 was completely abolished (Figure 5(ii)), demonstrating the dependence of [Ca^2+^]_CYT_ increases on ORAI1. Finally, we further validated the role of ORAI1 on AKT-mediated Ca^2+^ influx using ORAI1 siRNA in WT-GCaMP6m-MDA-MB-231 cells (Figure S2A). Consistent with results observed with ORAI1KO cells, siORAI1 also suppressed SC79-mediated Ca^2+^ influx in WT cells (Figure S2B). We also confirmed via immunoblot that both P2Y6 receptor and AKT2 expression was unaltered in *ORAI1*KO cells compared to WT cells (Figure S3A,B), showing that the suppressed SC79-induced Ca^2+^ influx in ORAI1*KO* cells is not due to a downregulation of purinergic receptor nor AKT. These results using both CRISPR/Cas9 and siRNA-mediated ORAI1 silencing confirm that AKT-mediated Ca^2+^ influx occurs through ORAI1.

**Figure 6:**
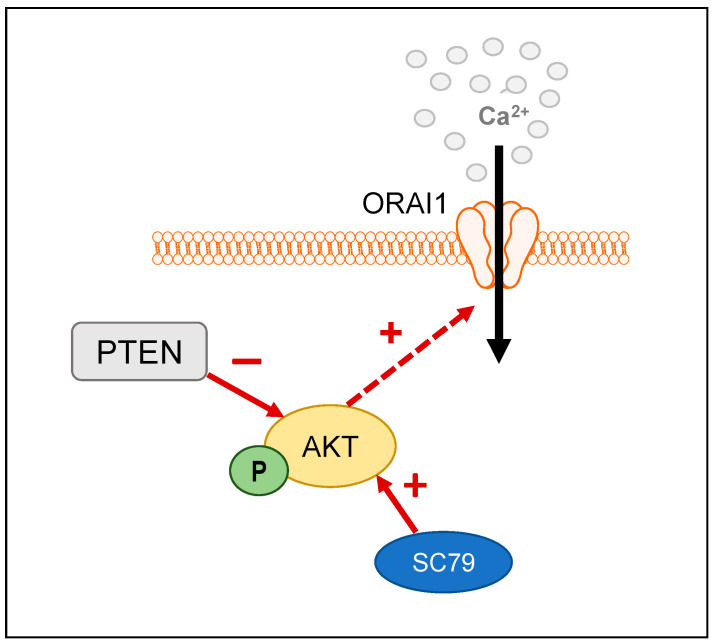
Graphical schematic summarizes the regulation of ORAI1-mediated Ca^2+^ influx by AKT. Increased AKT phosphorylation as a result of either PTEN silencing or pharmacological activation with SC79, results in increased Ca^2+^ influx through ORAI1 channels.

To date, many studies suggest that increases in Ca^2+^ influx can promote the activation of the PI3K/Akt pathway in cancer cells [21,22,41,42]. However, whether AKT activity can alter Ca^2+^ influx has remained unclear. In A431 carcinoma cells, pharmacological inhibition of AKT reduces SOCE [43], suggesting that changes in Ca^2+^ signaling may also occur as a result of AKT activity. Collectively, our results demonstrate that activation of AKT as a consequence of both PTEN silencing and direct pharmacological activation promotes Ca^2+^ influx through the ORAI1 calcium channel (Figure 6). Our results also implicate a role for the AKT2 isoform in this mechanism. These observations are significant since both ORAI1 and AKT2 are commonly involved in cancer cell migration and invasion [5,15,32,39,41].

Our findings that PTEN silencing and AKT activation promoted Ca^2+^ influx were counter-intuitive to reports that phosphorylated AKT reduces endoplasmic reticulum Ca^2+^ release [17,18]. It would be expected that reducing Ca^2+^ release would lower the driving force for Ca^2+^ influx to refill the endoplasmic reticulum Ca^2+^ stores. Therefore, it is possible that AKT-mediated Ca^2+^ influx through ORAI1 occurs through a store-independent mechanism. In HCT116 colon cancer cells, EGF stimulation increases AKT activation and in turn promotes STIM1 phosphorylation [41]. This results in increased Ca^2+^ influx through the formation of a TRPC1/ORAI1/SK3 complex [41]. Increased AKT phosphorylation due to insulin stimulation also increases ORAI1 trafficking to the plasma membrane in podocytes [44], where AKT2 is the predominant isoform [45]. Alternatively, AKT may also regulate ORAI1-mediated Ca^2+^ influx through more indirect pathways. In this regard, AKT and the serum- and glucocorticoid-inducible kinase 1 (SGK1) both share similar structures and function and are both substrates of the upstream kinase PDK1. Increased SGK1 expression enhances ORAI1-dependent Ca^2+^ influx via preventing NEDD,3,4-mediated degradation [46] or increasing ORAI1 expression through NF-κB [47]. It is possible that AKT could indirectly regulate the activity of ORAI1 via direct interactions and/or binding to SGK1. Indeed, SGK1 forms multimeric protein complexes with both Akt1 and Akt2 in C. elegans [48]. Further studies will be required to confirm if this phenomenon occurs in breast cancer cells and the consequences on Ca^2+^ signaling.

This study has some limitations. Firstly, our initial investigation into the link between increased AKT activity and ORAI1-mediated Ca^2+^ influx was done using a high throughput assay with the genetically encoded GCaMP6m sensor. Future studies could further characterize this initial modest Ca^2+^ phenotype as a result of increased AKT activation via single cell imaging to identify changes in calcium oscillations. These studies could incorporate the use of newer, genetically encoded ratiometric Ca^2+^ sensors such as mScarlet [49] or ratiometric dyes to better quantify changes in basal Ca^2+^. Secondly, the link between increased AKT activity and ORAI1-mediated Ca^2+^ influx should be investigated in more cell lines. Previous studies in our laboratory have already shown that MDA-MB-468 cells, which are PTEN-deficient and have increased AKT activity also exhibit increased basal Ca^2+^ influx [25]. Future studies should investigate this phenomenon in other breast cancer cell lines with high basal levels of phosphorylated AKT, such as SKBR3 cells [50]. Finally, there is a potential for off-target effects as a high siRNA (100 nM) concentration was used to achieve optimal AKT1 and AKT2 silencing in our study. As such, further studies exploring the role of AKT2 in SC79-mediated Ca^2+^ influx should optimize for AKT2 isoform-specific knockout such as the use of CRISPR/Cas9 gene knockout.

## 4. Conclusions

Our study showed that both PTEN knockdown and AKT pharmacological activation increased Ca^2+^ influx in MDA-MB-231 breast cancer cells. This Ca^2+^ influx is regulated partially by AKT2 and is mediated through ORAI1 calcium channels. Future studies assessing the mechanism by which increased AKT activation enhances ORAI1-mediated Ca^2+^ influx will further define the role of ORAI1 as a therapeutic target in cancers with AKT hyperactivation.

## Data Availability

Published and supporting data are available upon request.

## Supplementary Materials

The following supporting information can be downloaded at: https://www.mdpi.com/article/10.3390/cancers14194794/s1, Figure S1: PTEN silencing has no effect on CPA-induced Ca^2+^ increase in GCaMP6m-MDA-MB-231 cells. (A) (i) shows the representative Ca^2+^ trace as a result of CPA addition in GCaMP6m-MDA-MB-231 cells transfected with siNT or siPTEN. (ii) Bar graph compares the mean ± S.E.M. of the peak relative [Ca^2+^]_CYT_ increase in siNT and siPTEN transfected cells. Statistical analysis was done using a paired t-test. ns, not significant. Figure S2: ORAI1 silencing suppresses SC79-induced increases in Ca^2+^ influx in GCaMP6m-MDA-MB-231 cells. (A) shows ORAI1 protein levels after ORAI1 silencing (siORAI1). (B) (i) Representative trace shows the effect of siORAI1 on Ca^2+^ influx in GCaMP6m-MDA-MB-231 cells pre-incubated with SC79 (3 µM) for 1 h. (ii) Bar graph shows mean ± S.E.M. of the peak relative Ca^2+^ increase as a result of SC79 (3 µM) treatment. Statistical analysis was done using a paired t-test. ** *p* < 0.01. (iii) Concentration-response curve shows the effect of siORAI1 on the area under the curve of SC79-induced Ca^2+^ influx. Statistical analysis was done using a two-way ANOVA with a Bonferroni’s test. *** *p* < 0.001, ** *p* < 0.01, * *p* < 0.05. Figure S3: P2Y6 receptor and AKT2 protein levels are not altered in ORAI1*KO* cells. A(i) and B(i) Representative immunoblots showing the expression levels of P2Y6 receptor and AKT2 in *ORAI1*KO cells compared to WT cells. (ii) Bar graphs compare the mean ± S.E.M. of P2Y6 and AKT2 expression normalized to β-actin in *ORAI1*KO and WT cells. Statistical analysis was done using a paired t-test. ns, not significant. Figure S4: Uncropped Western blots for Figure 2, Figure 3, Figure 4 and Figure S3.

## References

[1] DeSantis C.E., Ma J., Gaudet M.M., Newman L.A., Miller K.D., Goding Sauer A., Jemal A., Siegel R.L. (2019). Breast cancer statistics, 2019. CA Cancer J. Clin..

[2] The Cancer Genome Atlas Network (2012). Comprehensive molecular portraits of human breast tumours. Nature.

[3] Cossu-Rocca P., Orrù S., Muroni M.R., Sanges F., Sotgiu G., Ena S., Pira G., Murgia L., Manca A., Uras M.G. (2015). Analysis of PIK3CA Mutations and Activation Pathways in Triple Negative Breast Cancer. PLoS ONE.

[4] Wang J., Zhao W., Guo H., Fang Y., Stockman S.E., Bai S., Ng P.K.-S., Li Y., Yu Q., Lu Y. (2018). AKT isoform-specific expression and activation across cancer lineages. BMC Cancer.

[5] Dillon R.L., Marcotte R., Hennessy B.T., Woodgett J.R., Mills G.B., Muller W.J. (2009). Akt1 and Akt2 Play Distinct Roles in the Initiation and Metastatic Phases of Mammary Tumor Progression. Cancer Res..

[6] Xing Y., Lin N.U., Maurer M.A., Chen H., Mahvash A., Sahin A., Akcakanat A., Li Y., Abramson V., Litton J. (2019). Phase II trial of AKT inhibitor MK-2206 in patients with advanced breast cancer who have tumors with PIK3CA or AKT mutations, and/or PTEN loss/PTEN mutation. Breast Cancer Res..

[7] Prevarskaya N., Ouadid-Ahidouch H., Skryma R., Shuba Y. (2014). Remodelling of Ca^2+^ transport in cancer: How it contributes to cancer hallmarks?. Philos. Trans. R Soc. Lond B Biol. Sci..

[8] Putney J.W. (1990). Capacitative calcium entry revisited. Cell Calcium..

[9] Hoth M., Penner R. (1992). Depletion of intracellular calcium stores activates a calcium current in mast cells. Nature.

[10] Soboloff J., Spassova M.A., Tang X.D., Hewavitharana T., Xu W., Gill D.L. (2006). Orai1 and STIM Reconstitute Store-operated Calcium Channel Function. J. Biol. Chem..

[11] Baldi C., Vazquez G., Boland R. (2003). Capacitative calcium influx in human epithelial breast cancer and non-tumorigenic cells occurs through Ca^2+^ entry pathways with different permeabilities to divalent cations. J. Cell. Biochem..

[12] McAndrew D., Grice D.M., Peters A.A., Davis F.M., Stewart T., Rice M., Smart C.E., Brown M.A., Kenny P.A., Roberts-Thomson S.J. (2011). ORAI1-Mediated Calcium Influx in Lactation and in Breast Cancer. Mol. Cancer Ther..

[13] Azimi I., Milevskiy M.J., Chalmers S.B., Yapa K.T., Robitaille M., Henry C., Baillie G.J., Thompson E.W., Roberts-Thomson S.J., Monteith G.R. (2019). ORAI1 and ORAI3 in Breast Cancer Molecular Subtypes and the Identification of ORAI3 as a Hypoxia Sensitive Gene and a Regulator of Hypoxia Responses. Cancers.

[14] Yang S., Zhang J.J., Huang X.-Y. (2009). Orai1 and STIM1 Are Critical for Breast Tumor Cell Migration and Metastasis. Cancer Cell.

[15] Ivanova H., Vervliet T., Monaco G., Terry L.E., Rosa N., Baker M.R., Parys J.B., Serysheva I.I., Yule D.I., Bultynck G. (2020). Bcl-2-protein family as modulators of IP3 receptors and other organellar Ca^2+^ channels. Cold Spring Harb. Perspect. Biol..

[16] Khan M.T., Wagner L., Yule D.I., Bhanumathy C., Joseph S.K. (2006). Akt kinase phosphorylation of inositol 1,4,5-trisphosphate receptors. J. Biol. Chem..

[17] Szado T., Vanderheyden V., Parys J.B., De Smedt H., Rietdorf K., Kotelevets L., Chastre E., Khan F., Landegren U., Söderberg O. (2008). Phosphorylation of inositol 1,4,5-trisphosphate receptors by protein kinase B/Akt inhibits Ca^2+^ release and apoptosis. Proc. Natl. Acad. Sci. USA.

[18] Marchi S., Corricelli M., Branchini A., Vitto V.A.M., Missiroli S., Morciano G., Perrone M., Ferrarese M., Giorgi C., Pinotti M. (2019). Akt-mediated phosphorylation of MICU1 regulates mitochondrial Ca(^2+^) levels and tumor growth. EMBO J..

[19] Feldman B., Fedida-Metula S., Nita J., Sekler I., Fishman D. (2010). Coupling of mitochondria to store-operated Ca^2+^-signaling sustains constitutive activation of protein kinase B/Akt and augments survival of malignant melanoma cells. Cell Calcium.

[20] Gocher A.M., Azabdaftari G., Euscher L.M., Dai S., Karacosta L.G., Franke T.F., Edelman A.M. (2017). Akt activation by Ca^2+^/calmodulin-dependent protein kinase kinase 2 (CaMKK2) in ovarian cancer cells. J. Biol. Chem..

[21] Han Y., Liu C., Zhang D., Men H., Huo L., Geng Q., Wang S., Gao Y., Zhang W., Zhang Y. (2019). Mechanosensitive ion channel Piezo1 promotes prostate cancer development through the activation of the Akt/mTOR pathway and acceleration of cell cycle. Int. J. Oncol..

[22] Sagredo A.I., Sagredo E.A., Cappelli C., Báez P., Andaur R.E., Blanco C., Tapia J.C., Echeverría C., Cerda O., Stutzin A. (2018). TRPM4 regulates Akt/GSK3-beta activity and enhances beta-catenin signaling and cell proliferation in prostate cancer cells. Mol Oncol..

[23] Azimi I., Milevskiy M.J.G., Kaemmerer E., Turner D., Yapa K.T.D.S., Brown M.A., Thompson E.W., Roberts-Thomson S.J., Monteith G.R. (2017). TRPC1 is a differential regulator of hypoxia-mediated events and Akt signaling in PTEN-deficient breast cancer cells. J. Cell Sci..

[24] Davis F.M., Peters A.A., Grice D.M., Cabot P.J., Parat M.-O., Roberts-Thomson S.J., Monteith G.R. (2012). Non-Stimulated, Agonist-Stimulated and Store-Operated Ca^2+^ Influx in MDA-MB-468 Breast Cancer Cells and the Effect of EGF-Induced EMT on Calcium Entry. PLoS ONE.

[25] So C.L., Meinert C., Xia Q., Robitaille M., Roberts-Thomson S.J., Monteith G.R. (2022). Increased matrix stiffness suppresses ATP-induced sustained Ca^2+^ influx in MDA-MB-231 breast cancer cells. Cell Calcium.

[26] Bassett J.J., Bong A.H., Janke E.K., Robitaille M., Roberts-Thomson S., Peters A.A., Monteith G.R. (2018). Assessment of cytosolic free calcium changes during ceramide-induced cell death in MDA-MB-231 breast cancer cells expressing the calcium sensor GCaMP6m. Cell Calcium.

[27] Schmidlin F., Amadesi S., Vidil R., Trevisani M., Martinet N., Caughey G., Tognetto M., Cavallesco G., Mapp C., Geppetti P. (2001). Expression and Function of Proteinase-activated Receptor 2 in Human Bronchial Smooth Muscle. Am. J. Respir. Crit. Care Med..

[28] Kuchay S., Giorgi C., Simoneschi D., Pagan J., Missiroli S., Saraf A., Florens L., Washburn M.P., Collazo-Lorduy A., Castillo-Martin M. (2017). PTEN counteracts FBXL2 to promote IP3R3- and Ca^2+^-mediated apoptosis limiting tumour growth. Nature.

[29] Marchi S., Marinello M., Bononi A., Bonora M., Giorgi C., Rimessi A., Pinton P. (2012). Selective modulation of subtype III IP3R by Akt regulates ER Ca^2+^ release and apoptosis. Cell Death Dis..

[30] Manning B.D., Toker A. (2017). AKT/PKB Signaling: Navigating the Network. Cell.

[31] Degan S.E., Gelman I.H. (2021). Emerging Roles for AKT Isoform Preference in Cancer Progression Pathways. Mol. Cancer Res..

[32] Hsu A.-L., Ching T.-T., Sen G., Wang D.-S., Bondada S., Authi K.S., Chen C.-S. (2000). Novel function of phosphoinositide 3-kinase in T cell Ca^2+^ signaling: A phosphatidylinositol 3, 4, 5-trisphosphate-mediated Ca^2+^ entry mechanism. J. Biol. Chem..

[33] Trebak M., Lemonnier L., DeHaven W.I., Wedel B.J., Bird G.S., Putney J.W. (2008). Complex functions of phosphatidylinositol 4,5-bisphosphate in regulation of TRPC5 cation channels. Pflügers Arch.-Eur. J. Physiol..

[34] Jo H., Mondal S., Tan D., Nagata E., Takizawa S., Sharma A.K., Hou Q., Shanmugasundaram K., Prasad A., Tung J.K. (2012). Small molecule-induced cytosolic activation of protein kinase Akt rescues ischemia-elicited neuronal death. Proc. Natl. Acad. Sci. USA.

[35] Danciu T.E., Adam R.M., Naruse K., Freeman M.R., Hauschka P.V. (2003). Calcium regulates the PI3K-Akt pathway in stretched osteoblasts. FEBS Lett..

[36] Catalucci D., Zhang D.-H., DeSantiago J., Aimond F., Barbara G., Chemin J., Bonci D., Picht E., Rusconi F., Dalton N.D. (2009). Akt regulates L-type Ca^2+^ channel activity by modulating Cav 1 protein stability. J. Gen. Physiol..

[37] Córdova-Casanova A., Olmedo I., Riquelme J.A., Barrientos G., Sánchez G., Gillette T.G., Lavandero S., Chiong M., Donoso P., Pedrozo Z. (2018). Mechanical stretch increases L-type calcium channel stability in cardiomyocytes through a polycystin-1/AKT-dependent mechanism. Biochim. Biophys. Acta Mol. Cell Res..

[38] Maroulakou I.G., Oemler W., Naber S.P., Tsichlis P.N. (2007). Akt1 ablation inhibits, whereas Akt2 ablation accelerates, the development of mammary adenocarcinomas in mouse mammary tumor virus (MMTV)-ErbB2/neu and MMTV-polyoma middle T transgenic mice. Cancer Res..

[39] Riggio M., Perrone M.C., Polo M.L., Rodriguez M.J., May M., Abba M., Lanari C., Novaro V. (2017). AKT1 and AKT2 isoforms play distinct roles during breast cancer progression through the regulation of specific downstream proteins. Sci. Rep..

[40] Zheng S., Zhou L., Ma G., Zhang T., Liu J., Li J., Nguyen N.T., Zhang X., Li W., Nwokonko R. (2018). Calcium store refilling and STIM activation in STIM- and Orai-deficient cell lines. Pflügers Arch.-Eur. J. Physiol..

[41] Guéguinou M., Harnois T., Crottes D., Uguen A., Deliot N., Gambade A., Chantôme A., Haelters J.P., Jaffrès P.A., Jourdan M.L. (2016). SK3/TRPC1/Orai1 complex regulates SOCE-dependent colon cancer cell migration: A novel opportunity to modulate anti-EGFR mAb action by the alkyl-lipid Ohmline. Oncotarget.

[42] Tang B.-D., Xia X., Lv X.-F., Yu B.-X., Yuan J.-N., Mai X.-Y., Shang J.-Y., Zhou J.-G., Liang S.-J., Pang R.-P. (2016). Inhibition of Orai1-mediated Ca^2+^entry enhances chemosensitivity of HepG2 hepatocarcinoma cells to 5-fluorouracil. J. Cell. Mol. Med..

[43] Emeriau N., De Clippele M., Gailly P., Tajeddine N. (2018). Store operated calcium entry is altered by the inhibition of receptors tyrosine kinase. Oncotarget.

[44] Kim J.-H., Hwang K.-H., Dang B.T.N., Eom M., Kong I.D., Gwack Y., Yu S., Gee H.Y., Birnbaumer L., Park K.-S. (2021). Insulin-activated store-operated Ca^2+^ entry via Orai1 induces podocyte actin remodeling and causes proteinuria. Nat. Commun..

[45] Canaud G., Bienaime F., Viau A., Treins C., Baron W., Nguyen C., Burtin M., Berissi S., Giannakakis K., Muda A.O. (2013). AKT2 is essential to maintain podocyte viability and function during chronic kidney disease. Nat. Med..

[46] Eylenstein A., Gehring E.M., Heise N., Shumilina E., Schmidt S., Szteyn K., Münzer P., Nurbaeva M.K., Eichenmüller M., Tyan L. (2011). Stimulation of Ca^2+^-channel Orai1/STIM1 by serum- and glucocorticoid-inducible kinase 1 (SGK1). FASEB J..

[47] Borst O., Schmidt E.-M., Münzer P., Schönberger T., Towhid S.T., Elvers M., Leibrock C., Schmid E., Eylenstein A., Kuhl D. (2012). The serum- and glucocorticoid-inducible kinase 1 (SGK1) influences platelet calcium signaling and function by regulation of Orai1 expression in megakaryocytes. Blood.

[48] Hertweck M., Göbel C., Baumeister R.C. (2004). elegans SGK-1 Is the Critical Component in the Akt/PKB Kinase Complex to Control Stress Response and Life Span. Dev. Cell.

[49] Zhang D., Redington E., Gong Y. (2021). Rational engineering of ratiometric calcium sensors with bright green and red fluorescent proteins. Commun. Biol..

[50] She Q.-B., Chandarlapaty S., Ye Q., Lobo J., Haskell K.M., Leander K.R., DeFeo-Jones D., Huber H.E., Rosen N. (2008). Breast Tumor Cells with PI3K Mutation or HER2 Amplification Are Selectively Addicted to Akt Signaling. PLoS ONE.

