# Supplementary material for: AKT Regulation of ORAI1-Mediated Calcium Influx in Breast Cancer Cells
Source: Cancers (Basel). 2022 Sep 30;14(19):4794. doi: 10.3390/cancers14194794 (PMC9562175; doi:10.3390/cancers14194794)
Supplement: Supplementary file 1 [file cancers-14-04794-s001.zip › cancers-1796837-supplementary.pptx]

## Slide 1
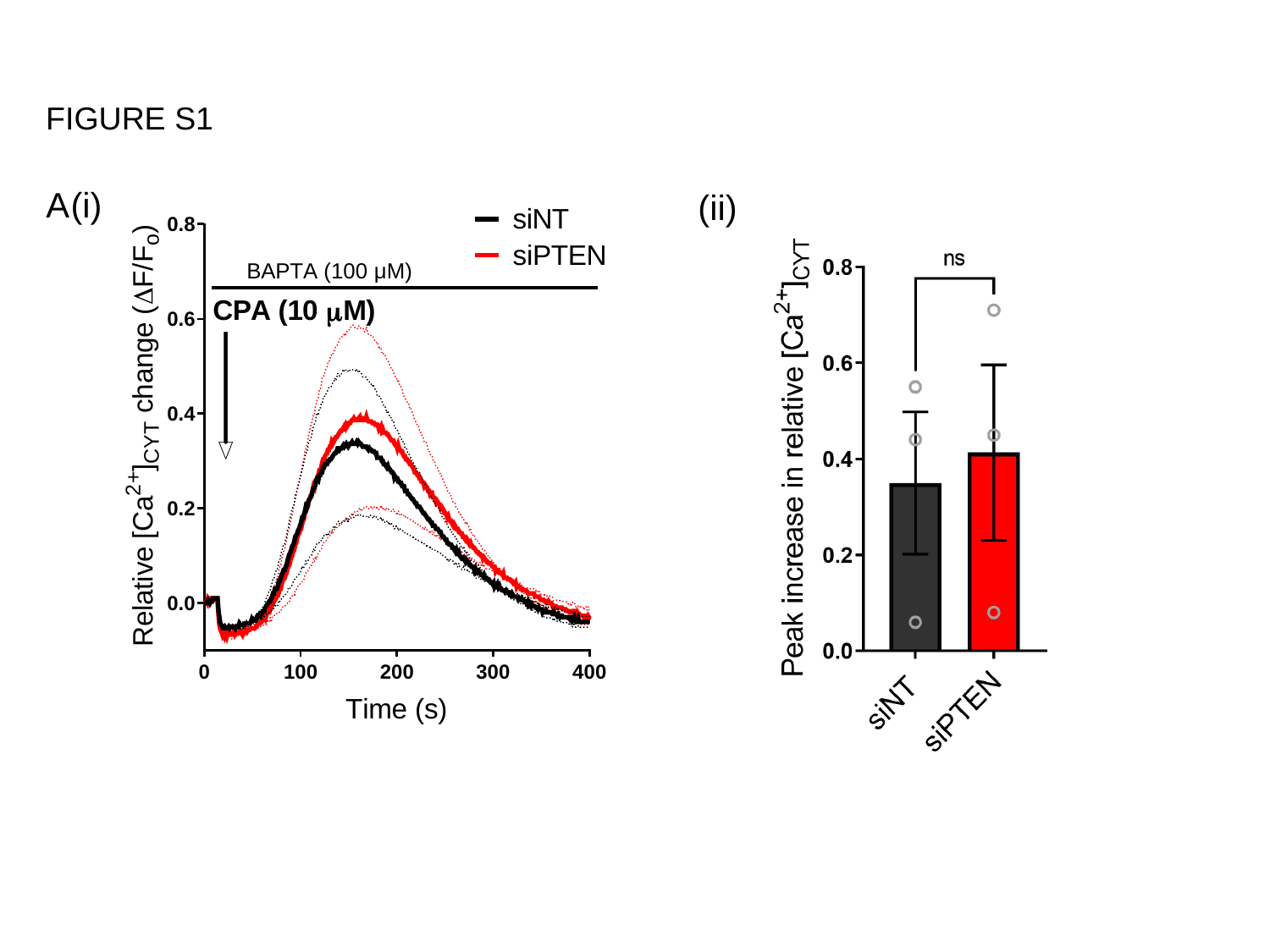

FIGURE S1
A
(i)
(ii)

## Slide 2
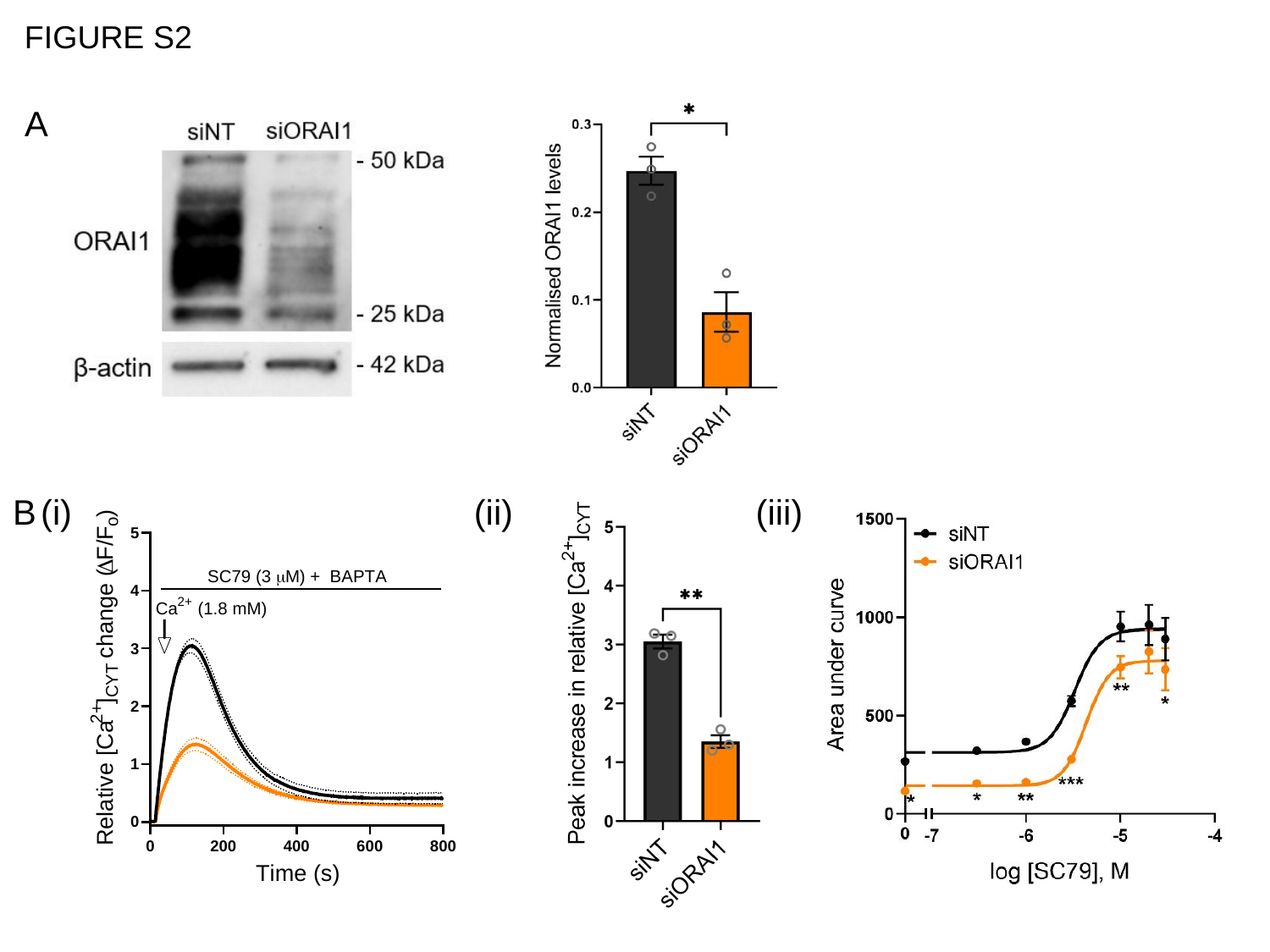

FIGURE S2
A
B
(i)
(ii)
(iii)

## Slide 3
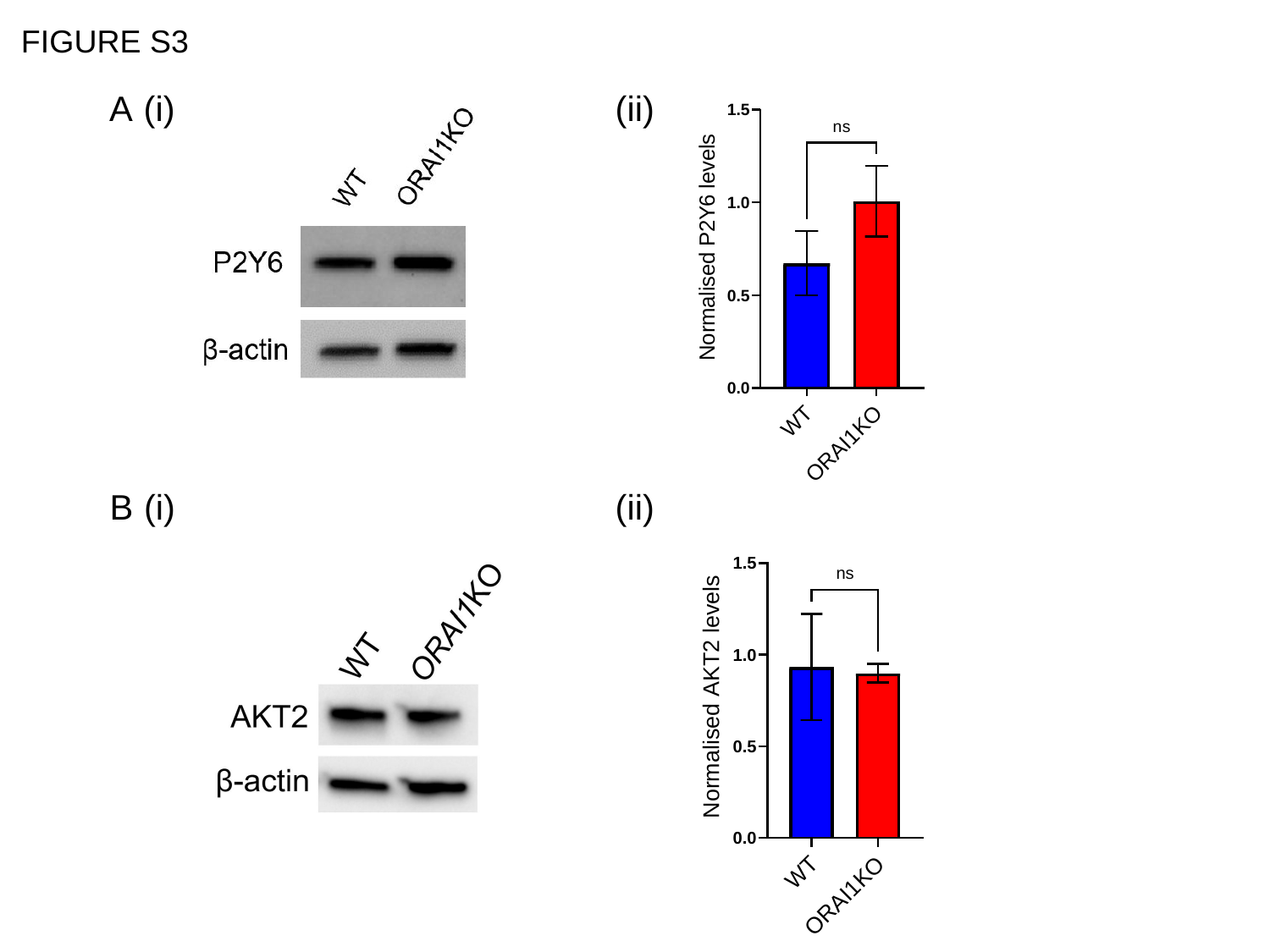

FIGURE S3
A
(i)
(ii)
B
(i)
(ii)

## Slide 4
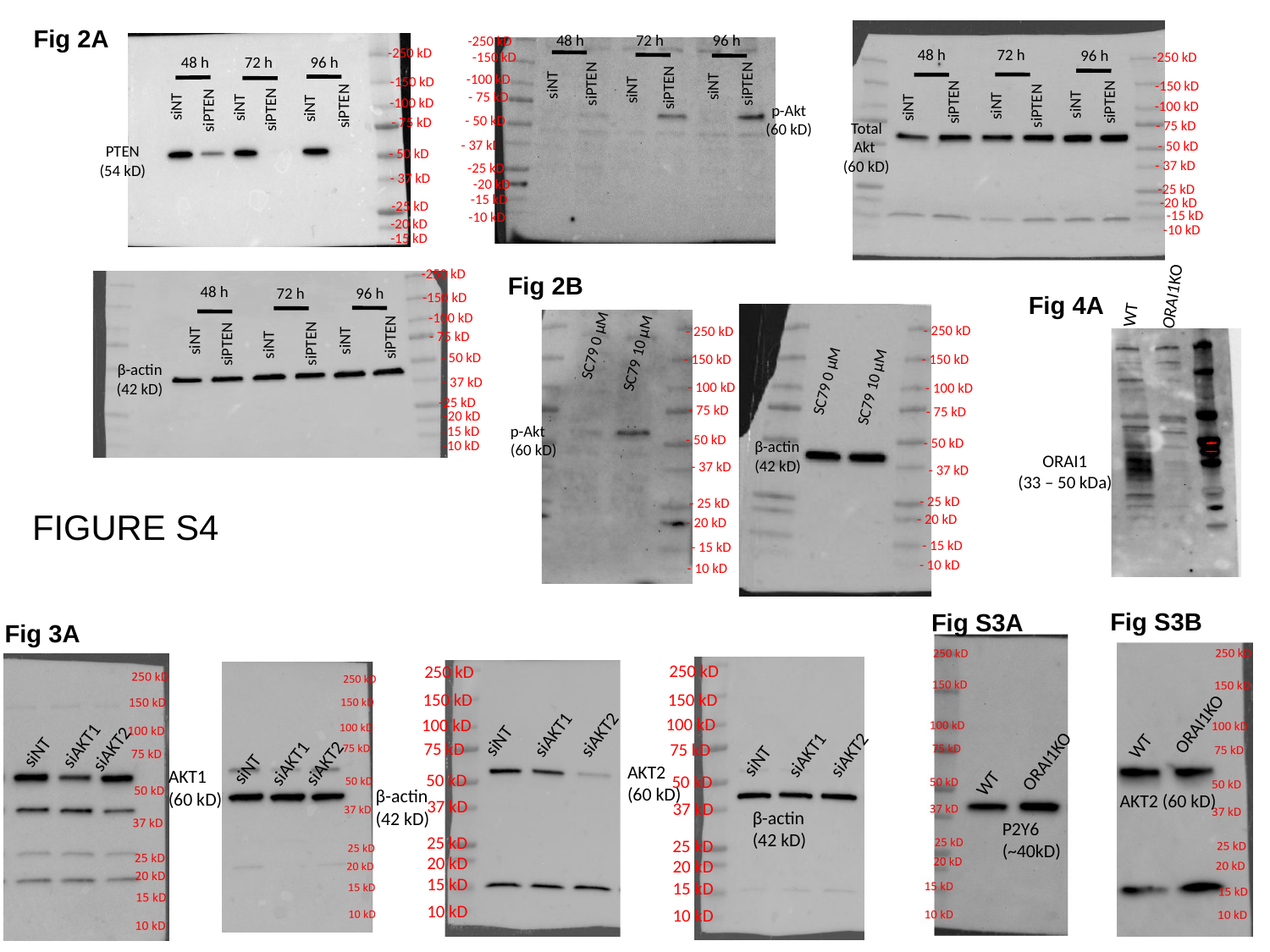

Fig 2A
48 h
72 h
96 h
-250 kD
-150 kD
siPTEN
siPTEN
siPTEN
siNT
siNT
-100 kD
siNT
- 75 kD
- 50 kD
- 37 kD
-25 kD
-20 kD
-15 kD
-10 kD
Total Akt
(60 kD)
48 h
72 h
96 h
-250 kD
-150 kD
siPTEN
siPTEN
-100 kD
siPTEN
siNT
siNT
siNT
- 75 kD
- 50 kD
-25 kD
-20 kD
-15 kD
-10 kD
p-Akt (60 kD)
-250 kD
96 h
48 h
72 h
-150 kD
siPTEN
siPTEN
siPTEN
-100 kD
siNT
siNT
siNT
- 75 kD
PTEN (54 kD)
- 50 kD
- 37 kD
-25 kD
-20 kD
-15 kD
- 37 kD
ORAI1KO
WT
ORAI1
(33 – 50 kDa)
-250 kD
48 h
96 h
72 h
-150 kD
-100 kD
siPTEN
siPTEN
siPTEN
- 75 kD
siNT
siNT
siNT
- 50 kD
β-actin (42 kD)
- 37 kD
-25 kD
-20 kD
-15 kD
-10 kD
Fig 2B
Fig 4A
- 250 kD
SC79 0 µM
SC79 10 µM
- 150 kD
- 100 kD
- 75 kD
p-Akt
(60 kD)
- 50 kD
- 37 kD
- 25 kD
- 20 kD
- 15 kD
- 10 kD
- 250 kD
- 150 kD
- 100 kD
- 75 kD
- 50 kD
β-actin
(42 kD)
- 37 kD
- 25 kD
- 20 kD
- 15 kD
- 10 kD
SC79 0 µM
SC79 10 µM
FIGURE S4
Fig S3B
Fig S3A
Fig 3A
siAKT1
siAKT2
siNT
250 kD
150 kD
100 kD
75 kD
siAKT2
siAKT1
siNT
50 kD
37 kD
β-actin
(42 kD)
25 kD
20 kD
15 kD
10 kD
250 kD
150 kD
100 kD
siAKT2
siAKT1
siNT
75 kD
AKT2
(60 kD)
50 kD
37 kD
25 kD
20 kD
15 kD
10 kD
siAKT2
siAKT1
siNT
ORAI1KO
WT
ORAI1KO
AKT1
(60 kD)
WT
β-actin
(42 kD)
AKT2 (60 kD)
P2Y6
(~40kD)
